# Beyond Motor Decline in ALS: Patient-Centered Insights into Non-Motor Manifestations

**DOI:** 10.3390/medicina61091694

**Published:** 2025-09-18

**Authors:** Anca Moțățăianu, Sebastian Andone, Smaranda Maier, Rareș Chinezu, Medeea Roman, Mihai Dumitreasă, Rodica Bălașa, Ioana Ormenișan

**Affiliations:** 1 Department of Neurology, University of Medicine, Pharmacy, Science and Technology of Târgu Mureș ‘George Emil Palade’, 540142 Târgu Mureș, Romania; 2 Neurology Clinic, Emergency Clinical County Hospital Târgu Mureș, 540136 Târgu Mureș, Romania; 3 Department of Neurosurgery, University of Medicine, Pharmacy, Science and Technology of Târgu Mureș ‘George Emil Palade’, 540142 Târgu Mureș, Romania

**Keywords:** amyotrophic lateral sclerosis, motor neuron disease, non-motor symptoms, autonomic dysfunction, frontotemporal dysfunction, sleep disorders, affective symptoms

## Abstract

*Background and Objectives*: Traditionally regarded as a purely motor disorder, amyotrophic lateral sclerosis (ALS) is a progressive neurodegenerative disease characterized by the degeneration of upper and lower motor neurons. However, it is increasingly recognized as a condition with a broader clinical spectrum, encompassing a variety of non-motor symptoms (NMS) that significantly impact patients’ quality of life and may influence disease progression and prognosis. *Materials and Methods*: The study included 44 patients diagnosed with probable or definite ALS and 35 healthy controls (HC). Functional neurological status, non-motor manifestations, and cognitive and affective domains were evaluated using the revised ALS Functional Rating Scale (ALSFRS-R), the Non-Motor Symptoms Questionnaire (NMSQuest), the Frontal Assessment Battery (FAB), and the Beck Depression Inventory (BDI), respectively. *Results*: A majority of ALS patients exhibited non-motor symptoms (NMS). Significant associations were identified between specific NMS domains and ALSFRS-R subdomains: sleep disturbances were associated with lower fine motor, respiratory, and total scores; digestive symptoms with lower bulbar, respiratory, and total scores; cardiovascular symptoms with lower total scores; urinary symptoms with higher bulbar subscores and a significantly slower progression rate (ΔPR); and sensory symptoms with higher gross motor subscores. BDI scores were negatively correlated with respiratory and bulbar functions, whereas FAB scores showed positive correlations with both bulbar and total ALSFRS-R scores. *Conclusions*: Non-motor symptoms are highly prevalent in this ALS cohort. These symptoms do not consistently correlate with greater motor impairment, as urinary and somatosensory involvement may occur independently of functional decline. Cognitive, affective, and behavioral alterations co-exist with motor symptoms and are associated with poorer overall functional performance.

## 1. Introduction

Amyotrophic lateral sclerosis (ALS) is a progressive neurodegenerative disorder primarily characterized by the degeneration of upper and lower motor neurons, leading to muscle weakness, atrophy, and ultimately respiratory failure. Traditionally considered a purely motor disease, increasing evidence over the past two decades has revealed a broader clinical spectrum, including a range of non-motor symptoms that significantly affect quality of life and may also influence disease progression and prognosis [1,2].

Recent studies have shown that cognitive impairment, depressive symptoms, and behavioral changes are common in ALS patients, yet these manifestations are often underrecognized in routine clinical care. Non-motor symptoms may present early in the disease course and include mood disorders, executive dysfunction, sleep disturbances, autonomic symptoms, and fatigue [3,4]. Cognitive and behavioral impairments—especially those affecting executive functions—are now understood to lie on a continuum with frontotemporal dementia, occurring in up to 50% of ALS cases [5]. Depression is another frequent comorbidity, with a prevalence ranging from 30% to 50%, and may influence both quality of life and survival [6,7].

Despite the growing recognition of these features, non-motor symptoms remain insufficiently explored in clinical research, particularly through standardized instruments. There is a lack of systematic evaluations using validated tools that can capture the breadth and severity of NMS in ALS. Furthermore, the prognostic significance of these symptoms is not yet fully understood.

In this study, we aimed to systematically assess non-motor symptoms in ALS patients using a combination of validated tools: the Non-Motor Symptoms Questionnaire (NMS Quest), the Beck Depression Inventory (BDI) for depressive symptoms, and the Frontal Assessment Battery (FAB) for evaluating executive dysfunction. We also explored the relationship between these symptoms and clinical characteristics, including their potential association with disease progression.

## 2. Materials and Methods

This observational, cross-sectional study included 44 patients that were diagnosed with ALS in the last 12 months in a tertiary care neurology center (Neurology Department of Mures University Clinical Emergency Hospital) [8]. The patients were included in the study from 1 August 2022 to 1 November 2022. Additionally, we enrolled a control group of 35 healthy subjects.

All patients included in the study were diagnosed with probable or definite ALS according to the revised El Escorial criteria [8]. Exclusion criteria comprised the following: 1. diagnosis of other neurodegenerative diseases; 2. subjects whose Mini-Mental State Examination (MMSE) scores were <24; 3. comorbid conditions with potential impact on autonomic nervous system function, such as diabetes mellitus or glucose intolerance, vitamin deficiencies, impaired hepatic or renal function, endocrinopathies, autoimmune disorders, and heart failure; and 4. treatment with medication potentially affecting the autonomic nervous system or patients on sedatives, antidepressants, or anxiolytics.

### 2.1. Neurological Assessment

At baseline, each patient underwent a complete neurological and neurophysiological examination evaluating disease severity and progression. The functional neurological status was assessed by using the revised ALS Functional Rating Scale (ALSFRS-R) [9,10]. The ALSFRS-R assessment included four subscores calculated for bulbar function (ALSFRS-R-B), lower limb function (ALSFRS-R-LL), upper limb function (ALSFRS-R-UL), and respiratory function (ALSFRS-R-R), with a maximum total score of 48 points [9]. The initial ALSFRS-R scores were recorded at the time of diagnosis, and the second one was recorded at the baseline visit. The ALSFRS-R progression rate (ΔPR) was calculated as the change in ALSFRS-R score from diagnosis to the date of study visit, using the following formula: 48 − [(ALSFRS-R at diagnosis − ALSFRS-R at study visit)/duration of symptoms (in months)] [11].

At the time of diagnosis, patients were categorized into three distinct ALS phenotypes based on clinical presentation and electromyographic findings: 1. classical or typical ALS, characterized by predominant lower motor neuron (LMN) signs in at least two body regions, accompanied by upper motor neuron (UMN) involvement in at least one region; 2. LMN-predominant ALS, encompassing phenotypic variants such as flail arm and flail leg syndromes; and 3. bulbar-onset ALS, defined by the early and progressive manifestation of bulbar symptoms, including dysarthria and dysphagia [12]. Patients were also classified into spinal or bulbar ALS subtypes based on the site of symptom onset. The spinal subtype included cases with initial limb weakness (upper or lower), while the bulbar subtype was defined by the early presence of bulbar symptoms such as dysarthria or dysphagia.

The ALS progression pattern was evaluated by the extension of muscle atrophy or weakness beyond the initial site of symptom onset, based on clinical examination, electrophysiological studies, and patient history. Patients were subsequently classified into two groups according to the direction of motor neuron degeneration: 1. a horizontal spreading pattern (HSP), defined by disease progression from the cervical region to the contralateral cervical region or from the lumbar region to the contralateral lumbar region; and 2. a vertical spreading pattern (VSP), characterized by disease extension from the cervical or lumbar region to the ipsilateral limb, from the bulbar region to the cervical or lumbar segments, or vice versa [13].

### 2.2. Evaluation of Non-Motor Symptoms

Non-motor symptoms were systematically assessed using the Non-Motor Symptoms Questionnaire (NMS Quest), a validated 30-item screening tool designed to identify a broad range of non-motor manifestations [14,15]. Although originally developed for Parkinson’s disease, it was employed in this study as a structured screening tool to capture the non-motor symptoms relevant to ALS.

The questionnaire is structured into few domains, including cognitive (memory deficits, attention/concentration deficit), psychiatric (apathy, sadness, anxiety, perceptual problems, hallucinations), sleep/fatigue (tiredness, insomnia, vivid dreams, sleep-talking), sensory (pain, discomfort in legs, leg swelling), cardiovascular (dizziness/lightheadedness, falls), urinary (urinary urgency, nocturia), sexual (interest in sex, sexual dysfunction), gastrointestinal (dribbling, difficulty swallowing, vomiting/nausea, constipation, bowel incontinence, incomplete bowel emptying, loss of/change in taste/smell), and miscellaneous(weight change, excessive sweating, double vision) [16]. In this study, the NMS Quest was administered through structured interviews conducted by a trained examiner. This approach ensured standardized data collection, minimized reporting bias, and allowed clarification of any ambiguous patient responses, including distinguishing falls due to weakness from falls caused by impaired cardiac autonomic control (i.e., orthostatic hypotension). The information obtained was used to determine the presence and distribution of non-motor symptoms across the cohort. Responses were analyzed for prevalence.

Prior to the NMS Quest interview, all participants received a document outlining the study’s objectives and ensuring the confidentiality of their data. Written informed consent was obtained from each participant, in accordance with the protocol approved by the hospital’s ethics committee, before any data were recorded in the study database.

### 2.3. Neuropsychological Assessment

Cognitive and affective functions were assessed using the Frontal Assessment Battery (FAB), the Beck Depression Inventory (BDI), and the Mini-Mental State Examination (MMSE). Executive function was assessed with the FAB, a brief neuropsychological tool designed to evaluate frontal lobe functions through six subtests, including conceptualization, mental flexibility, motor programming, sensitivity to interference, inhibitory control, and environmental autonomy [17,18].

Affective symptoms, particularly depressive features, were measured using the Beck Depression Inventory II (BDI-II), a validated self-report questionnaire comprising 21 items rated on a four-point scale that quantify the severity of depressive symptoms. BDI-II, the Romanian version, was completed by all the study participants. BDI-II assesses the presence and severity of the depressive symptoms and evaluates the cognitive, motivational, autonomic, and somatic domains [19].

The MMSE was administered as a global cognitive screening tool, evaluating orientation, registration, attention and calculation, recall, and language abilities [20]. All neuropsychological assessments were performed in a structured manner by trained examiners during the clinical evaluation. In this study, higher BDI scores were interpreted as indicative of more severe depressive symptoms, while lower FAB scores reflected greater executive dysfunction. BDI and FAB scores obtained by the ALS patients were compared to those in the HC population enrolled. Subsequent correlation analyses were performed to explore the relationship between these neuropsychological variables and patients’ functional status, as measured by the ALSFRS-R. The interpretation of FAB and BDI scores was integral to the evaluation of the interplay between mood, cognitive decline, and physical disability in ALS. FAB and BDI allowed a structured evaluation of cognitive and affective domains, which are both recognized as integral to the broader spectrum of NMS in ALS.

### 2.4. Statistical Analysis

Descriptive statistics were performed on the demographics, clinical characteristics, and non-motor symptoms of the ALS patient cohort. Continuous variables such as age and ALSFRS-R scores were expressed as means ± standard deviations (SD), while categorical variables, including ALS subtype, phenotype, and non-motor symptoms, were reported as frequencies and percentages.

The impact of non-motor symptoms on the ALS Functional Rating Scale-Revised (ALSFRS-R) subscores—respiratory, bulbar, fine motor, and gross motor functions—as well as on the disease progression rate (ΔPR), was evaluated using ANOVA test, with statistical significance set at *p* < 0.05.

Correlation analyses were conducted using Pearson’s correlation coefficient to investigate relationships between neuropsychological measures and functional status. Statistical significance for correlations was set at *p* < 0.05.

We used Shapiro–Wilk to test the normality of the continuous variables. As distributions did not significantly deviate from normality, we chose ANOVA to compare functional scores between groups.

To evaluate the relationship between non-motor symptoms and functional status in ALS patients, an ANOVA analysis was conducted. Given the exploratory nature of the study and the relatively small sample size, *p*-values were reported without correction for multiple comparisons. As such, the results should be interpreted cautiously and considered hypothesis-generating.

All statistical tests and analyses were using IBM SPSS Statistics, Version 26 (IBM Corp., Armonk, NY, USA). Tables and figures were created using Microsoft Excel 2019 (Microsoft Corp., Redmond, WA, USA).

## 3. Results

### 3.1. Demographics and Clinical Characteristics of the Study Participants

HC and patient demographics and clinical characteristics, including ALSFRS-R scores at the time of the study visit are detailed in Table 1.

### 3.2. Description of Non-Motor Symptoms

A wide range of non-motor manifestations was reported, reflecting the multisystem involvement of ALS. Cognitive symptoms were identified in 7 patients (15.9%), including memory impairment and attention deficits. Psychiatric symptoms were observed in 28 patients (63.3%), most commonly apathy, depression, anxiety, and perceptual disturbances. Sleep disorders were highly prevalent, affecting 35 patients (79.5%), and included insomnia, vivid dreams, and sleep-talking. Sensory symptoms were reported by 37 patients (84%), such as pain, discomfort in the lower limbs, and sensations of leg swelling. Cardiovascular symptoms, including dizziness and light-headedness, were noted in 30 patients (68.2%). Genitourinary complaints were present in 32 patients (72.7%) and consisted of urinary urgency, nocturia, and sexual dysfunction. Gastrointestinal symptoms were reported by 36 patients (81.8%) and included dribbling, dysphagia, vomiting, constipation, and bowel incontinence. The Non-Motor Symptoms Questionnaire results per domain are summed up in Table 2.

### 3.3. Association Between Non-Motor Symptoms and Functional Status in ALS Patients Assessed by ALSFRS-R Scores and Disease Progression Rate

To evaluate the relationship between non-motor symptoms and functional status in ALS patients, an ANOVA analysis was conducted using ALSFRS-R subdomain scores and ΔPR as outcome variables, as shown in Table 3 below. Significant associations were identified for several non-motor symptom categories. Patients with sleep disorders had significantly lower ALSFRS-R fine motor scores (*p* = 0.014) and total scores (*p* = 0.008), along with a reduced respiratory subscore (*p* = 0.030), suggesting a more pronounced functional decline. Similarly, the presence of digestive symptoms was associated with significantly lower bulbar (*p* = 0.026) and respiratory (*p* = 0.044) subscores, as well as lower ALSFRS-R total scores (*p* = 0.013). Cardiovascular symptoms were also linked to lower ALSFRS-R total scores (*p* = 0.027). Interestingly, urinary symptoms were associated with higher bulbar subscores (*p* = 0.012) and a significantly slower progression rate (ΔPR) (*p* = 0.045). Additionally, patients with sensory symptoms had significantly higher gross motor subscores (*p* = 0.039). Other non-motor symptoms, such as psychiatric symptoms, cognition/behavior changes, and sexual dysfunction, did not show statistically significant differences in ALSFRS-R scores or ΔPR.

### 3.4. Correlation Between Affective Symptoms, Frontal Executive Function, and Functional Status in ALS Patients

The correlation analysis between the Beck Depression Inventory (BDI), Frontal Assessment Battery (FAB), and ALSFRS-R scores revealed significant associations. Negative correlations were observed between BDI scores and both respiratory (r = −0.217, *p* = 0.023) and bulbar (r = −0.209, *p* = 0.028) subscores of ALSFRS-R, indicating that higher depressive symptoms were linked to lower functional performance in these areas. A more pronounced negative correlation was found with the fine motor subscore (r = −0.430, *p* < 0.0001) and total ALSFRS-R score (r = −0.422, *p* < 0.0001), suggesting that affective symptoms interrelate closely with fine motor skills and overall disease severity. In contrast, the FAB showed a positive correlation with the bulbar (r = 0.363, *p* < 0.0001) and total ALSFRS-R scores (r = 0.339, *p* < 0.0001), indicating that better frontal executive function was associated with better motor performance in these domains. However, no significant correlations were observed either between FAB and the respiratory, gross motor, or fine motor subscores, or between BDI and the gross motor subscore.

**Figure 1:**
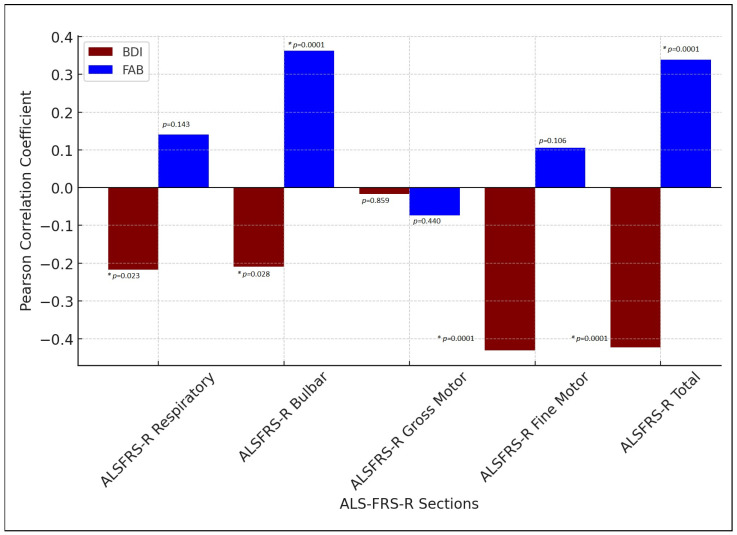
Pearson’s correlation analysis between BDI, FAB and ALSFRS-R scores. * Statistically significant correlation set at *p* < 0.05. BDI: Beck Depression Inventory. FAB: Frontal Assessment Battery.

The correlation matrix between BDI, FAB and ALSFRS-R scores is detailed in Figure 1.

## 4. Discussion

Recent evidence has increasingly recognized ALS as a multisystem neurodegenerative disorder. Although the motor system remains the primary area of involvement, there is growing acknowledgment of NMS in the disease’s clinical manifestation [21]. Impairments in the autonomic and sensory nervous systems, along with cognitive and psychiatric disturbances, contribute to the broader clinical profile of ALS patients. We acknowledge that there is currently no universally accepted system for categorizing NMS in ALS, as the field is still evolving. To facilitate interpretation and enhance clinical relevance, we grouped the original nine NMS Quest domains into four broader categories. We hereby propose four overarching NMS domains in ALS: autonomic dysfunction (encompassing gastrointestinal, genitourinary, cardiovascular dysfunction), sensory dysfunction, cognitive impairment and frontotemporal dysfunction, and respectively, sleep disturbances and affective symptoms.

### 4.1. Autonomic Dysfunction

Similar to other neurodegenerative diseases, like Parkinson’s disease, emerging evidence highlights the role of the gut–brain axis in ALS pathophysiology. Gut dysbiosis may contribute to neuroinflammation through immune activation, blood–brain barrier disruption, and aberrant protein accumulation (i.e., amyloid) [22]. In ALS, gut dysbiosis and increased intestinal permeability may facilitate neuroinflammation via immune activation and neurotoxin translocation, contributing to motor neuron degeneration. Microbiota-derived metabolites also appear to influence disease progression by modulating immune responses [23,24,25].

A 2022 genome-wide association study examining gut flora and ALS susceptibility identified a higher relative abundance of *Sutterella*, *Lactobacillales*, and elevated levels of gamma-glutamyl-phenylalanine—an intermediate in the gamma-glutamyl pathway—as being associated with increased ALS risk [26]. In transgenic SOD1^G93A^ mice, myenteric neurons exhibit aggregated pathological SOD1, leading to neuronal loss and impaired intestinal barrier integrity, marked by enteric glial activation and inflammatory cell infiltration. Notably, oral administration of a multistrain probiotic improved locomotor function and survival and reduced aberrant SOD1 accumulation in spinal cord neurons [27]. These findings support a bidirectional relationship between gut microbial dysbiosis and ALS pathogenesis, though causality remains unclear Future prospective studies including human models are needed.

Gastrointestinal dysfunction represents a relevant yet underrecognized feature of ALS. Several studies have examined digestive symptoms in relation to disease severity and phenotype, with constipation and early satiety frequently reported across clinical subtypes, irrespective of onset form [28]. In another study assessing upper gastrointestinal motor function in relation to ALS onset phenotype (bulbar vs. spinal) and disease severity, no significant correlation was observed between digestive symptoms—such as delayed gastric emptying or esophageal dysmotility—and ALSFRS-R scores or muscle strength measured by the Medical Research Council scale [29]. In our cohort, the majority of patients exhibited gastrointestinal symptoms—such as dribbling, difficulty swallowing, vomiting, constipation, bowel incontinence—and demonstrated significantly lower total ALSFRS-R scores, along with reduced respiratory and bulbar subscores, compared to the asymptomatic group. In addition, a high prevalence of constipation has been reported in the literature, several studies highlighting its association with lower functional scores. One such study further identified constipation as an independent risk factor for survival in patients with ALS [30].

Urinary dysfunction in ALS includes symptoms like incontinence, urgency, nocturia, and incomplete bladder emptying. Several studies confirmed the presence of urinary dysfunction among ALS patients. Given the interplay of supra-spinal lesions, spasticity and low mobility, there is still a debate regarding its pathophysiology. Urodynamic findings suggest that neurogenic bladder, with an overactive detrusor and high urethral resistance due to a contracted external sphincter, is the cause of urinary impairment [31]. Furthermore, neuropathological studies showed gliosis of the periaqueductal gray (PAG) and striatum of a 75-year-old ALS patient, both structures controlling micturition and defecation [32]. Urinary retention in these patients was also hypothesized to be associated with spasticity of the pelvic floor muscles, Lopes et al. finding a correlation between the Ashworth Scale, measured on the lower limbs, and the bladder post-void residual [33].

The relationship between urinary symptoms and ALS clinical status or disease progression remains inconsistent across studies. One of such studies reported no significant association between the clinical profile—phenotype, disease severity, time from symptom onset—and lower urinary tract or neurogenic bladder symptoms. In contrast, findings from Dubbioso’s study indicate that urinary complaints were an independent predictor of shorter survival [28,34]. Interestingly, in our ALS cohort, patients presenting with urinary symptoms exhibited slower rates of disease progression, and demonstrated higher bulbar subscores. However, this univariate analysis does not control for potential confounders, like age. Further multivariate analyses and longitudinal studies are warranted to better understand the complex interplay between NMS and functional impairment in ALS.

Conversely, no significant difference was found for the sexual domain in our study. This result agrees with the findings of several other studies investigating this domain in ALS populations [28,35]. Even though sexuality-related issues are common among ALS patients, a previous study reported that more than 75% of clinicians were unaware of any strategies or interventions to support their patients in this regard [36].

Cardiovascular dysregulation is a common feature in neurodegenerative diseases. In ALS, impaired cardiovascular neural regulation has been reported, presenting with palpitations, dizziness, light-headedness, and falls [37]. Neurally mediated cardiovascular disorders, such as orthostatic and postprandial hypotension or vasovagal syncope, are potential contributors to falls and associated morbidity and mortality in ALS patients [38].

The underlying pathophysiological processes proposed to date in ALS patients include reduced baroreceptor sensitivity, cardiac sympathetic hyperactivity at rest [39], or parasympathetic deterioration [37]. A decrease in the vagal tone and autonomic imbalance has been observed in ALS patients, where parasympathetic hypoactivity was demonstrated in ALS patients with bulbar signs [40]. Parasympathetic impairment in ALS patients was documented by several other studies [4,28,41].

Dizziness, lightheadedness or falls are symptoms pertaining to orthostatic hypotension (OH), which is another indicator of autonomicreflex failure. In a large ALS population, Quarracino et al. showed that out of the 1240 patients included, 10% presented with neurogenic OH, which is higher than the prevalence reported in the healthy middle-aged population [42].

More than half of our patients presented with cardiovascular symptoms. Dizziness, lightheadedness and falls were reported by 30 out of the 44 patients included. In addition, significantly lower total ALSFRS-R scores were recorded in this group compared to the patients without cardiovascular complaints. In agreement with other authors, we emphasize the need to address these dysautonomia symptoms as risk factors for cardiovascular events entailing increased morbidity and mortality. Given the supporting evidence that subclinical cardiac autonomic control dysfunction may be present early in the course of the disease [37], and that a decrease in life expectancy in ALS patients is due to cardiovascular collapse or sudden cardiac death [43], focused cardiovascular workup or cardiac function monitoring of ALS patients becomes more and more relevant.

### 4.2. Sensory Dysfunction

Despite its probable role in bulbar dysfunction, impaired dexterity and gait, somatosensory involvement is often neglected in motor neuron diseases. Clinical care is solely focused on the critical aspects of ALS, namely the preservation of motor independence and managing respiratory and bulbar symptoms [44], ignoring the large proportion of ALS patients who report pain or minor sensory symptoms throughout the course of the disease [45].

From the available clinical, imaging, histopathology or neurophysiology data, the literature provides vast evidence that the somatosensory system is not spared [44]. The majority of our ALS patients presented with sensory symptoms (pain, discomfort in legs, swollen legs). Interestingly, they had significantly higher scores on the gross motor ALSFRS-R subscore. We can safely infer that these symptoms are not due to reduced mobility or physical inactivity following severe physical impairment. Furthermore, several studies documented the presence of sensory signs and symptoms early in the course of the disease, sometimes as a subclinical finding, without significant concomitant motor complications [46,47].

Following skin biopsy and nerve conduction studies, sensory system impairment in ALS has been well documented in the literature [46,48,49,50,51,52,53,54,55]. In the European multicenter ESTEEM study, 20 of 88 ALS patients (22.7%) demonstrated sensory nerve conduction abnormalities, including reduced velocities and action potential amplitudes [48]. To assess large fiber sensory involvement, distal (antidromic dorsal sural and orthodromic medial plantar) and conventional (unilateral median and bilateral sural) sensory nerve conduction studies (NCS) were performed in 18 ALS patients and 31 controls. Abnormal amplitude and velocity values were found in 44.4% of patients on conventional NCS, and in 66.7% on distal NCS [50].

Multiple levels of the sensory pathway appear to be affected in ALS. Vaughan et al. reported degeneration of proprioceptive nerve endings at muscle spindles in ALS mouse models [52]. Additionally, satellite glial cells were identified as early targets of cytoplasmic mutant SOD1^G93A^ accumulation in presymptomatic mice, suggesting a neurotoxic mechanism contributing to primary sensory alterations [53]. In a study using skin punch biopsies from 18 ALS patients and 18 controls, Ren Y. et al. found TDP-43 deposition in all samples, while phosphorylated TDP-43 (pTDP-43) was detected in 33.3% of ALS cases and correlated with higher Small-Fiber Neuropathy Symptoms Inventory Questionnaire (SFN-SIQ) scores, implicating small fiber involvement [54]. Additionally, cultured sensory neurons from ALS mouse models expressing TDP43^A315T^ or SOD1^G93A^ showed impaired neurite outgrowth compared to wild-type controls, supporting the presence of sensory neurodegeneration in ALS [55].

There are numerous neuroimaging studies backing sensory involvement in ALS, demonstrating reduced gray matter volume in the left postcentral gyrus and corticospinal tract demyelination at the cervical level in spinal onset ALS [56,57], gray matter atrophy in the left postcentral gyrus and glucose hypometabolism in the bilateral thalamus [58] or even spinal cervical cord gray and white matter atrophy [59].

Given its progressive nature and the aggregation and propagation of the specific SOD1 and TDP-43 proteins, a prion-like mechanism underlying the ALS pathophysiology has been recently promoted in the literature [60], defining ALS as a process starting focally and progressing to contiguous anatomic regions, affecting non-motor neurons [40]. There are authors suggesting that patient groups with sensory nerve dysfunction may represent a variant of ALS and that it may be part of the primary disease [48]. What becomes increasingly obvious is that abnormal sensory findings are compatible with the diagnosis of ALS and that they are not solely indicative of ALS-Plus or ALS-Mimic syndromes [8].

### 4.3. Cognitive Impairment and Frontotemporal Dysfunction

Acknowledging ALS as a multisystem disease that extends beyond motor neurons degeneration, the presence of cognitive and behavioral changes was demonstrated by numerous studies [61]. It is now recognized that frontotemporal dysfunction (FTD) accompanies motor symptoms in ALS. Cognitive impairment in ALS is clinically characterized by behavioral disinhibition, poor judgment, impairments in executive function, verbal praxis and immediate recall [62]. Furthermore, frontotemporal dementia and ALS, which lie on a shared clinical and pathological spectrum, exhibit overlapping genetic and neuropathological features [63]. Both are associated with TDP-43 dysregulation and mutations in genes involved in lysosomal and autophagy pathways [63,64].

The term frontotemporal spectrum disorder (ALS-FTSD) is proposed to better reflect the complexity of FTD. According to the Strong criteria, a diagnosis of ALS-FTD requires progressive cognitive and/or behavioral decline with loss of insight and/or psychotic features, or language impairment consistent with semantic dementia or non-fluent variant primary progressive aphasia. In the absence of full FTD criteria, cognitive or behavioral changes are classified as ALS with cognitive impairment (ALSci) or ALS with behavioral impairment (ALSbi). Furthermore, the ALS–dementia variant includes patients developing dementia not pertaining to FTD [65]. The FAB proved to be a reliable and accurate tool in detecting frontal lobe dysfunction in ALS patients [66].

More than 50% of ALS patients exhibit changes in language, behavior, memory, or social cognition [67]. Common cognitive deficits include impaired attention, concentration, verbal fluency, and FTD-spectrum cognitive impairment [68]. Increasing evidence indicates that cognitive and behavioral changes may accompany or even precede motor symptoms [69], with some studies suggesting a parallel decline of motor and cognitive functions [70]. Moreover, bulbar-onset ALS has been associated with a higher risk of cognitive impairment [71].

In our study, the time interval from disease onset to disease diagnosis was longer in patients presenting with cognitive impairment. Furthermore, we found moderate positive correlations of the FAB scores with the bulbar dysfunction and respectively, the global motor dysfunction. Deterioration of the physical functions may thus occur alongside the cognitive and behavioral changes pertaining to frontal lobe dysfunction. Intriguingly, Benbrika S. et al. suggest that cognitive function may be preserved throughout the course of the disease in ALS patients presenting with intact cognition at disease onset [72].

Given that comorbid FTD is considered a negative prognostic factor in ALS [73], its correlation with delays in disease diagnosis, severity and progression remains to be established.

### 4.4. Sleep Disturbances and Affective Symptoms

Sleep disturbances are reported early in the course of neurodegenerative diseases and the relationship between the two is deemed bidirectional, poor sleep may both contribute to and result from disease progression [74]. Although poorly acknowledged, it is recognized that newly diagnosed ALS patients have poor sleep quality [75], disorders of sleep being reported by 50–63% of patients with ALS [76]. In a prospective cohort study conducted on 396,918 individuals from the UK Biobank, data showed that organic sleep disorders—insomnia, hypersomnia, sleep–wake rhythm disorders, and sleep apnea—were associated with an increased risk of ALS and ALS-FTSD [77]. Polysomnographic studies conducted in ALS patients revealed decreased sleep efficiency, reduced REM and slow-wave sleep, and increased wakefulness [78]. Hypothalamic atrophy and pTDP-43 deposition may underlie these disturbances [79]. REM sleep reduction may also impair amygdala function, potentially contributing to mood disorders [80].

Sleep disturbances—difficulty falling asleep, vivid dreams, sleep-talking—are a common finding in our study. Moreover, the patients complaining of disrupted sleep exhibited greater global and fine motor dysfunction. This finding is supported by another study showing that patients reporting poor sleep at baseline recorded lower functional scores [81]. Silva et al. suggest that sleep disruption—independent of respiratory events—and poor sleep quality are associated with reduced survival in ALS patients [82]. Further research is needed to clarify the role of sleep disturbances as a risk factor and their impact on disease progression.

The prevalence of depression in ALS patients ranges from 10% to 45% [83,84]. However, it is frequently underdiagnosed given the overlapping cognitive and behavioral changes that confound depressive symptoms in these patients [69]. Nonspecific features such as insomnia, hypersomnia, and fatigue—often linked to physical impairment—further complicate diagnosis [83]. While fatigue and depression may overlap, evidence suggests that fatigue is a distinct clinical entity in this population [85]. Anhedonia, a key feature of depression, and apathy, marked by reduced motivation and empathy, are both common in ALS. Apathy is included in ALSbi criteria and often overlaps with depressive symptoms [65,86,87]. The distinction between apathy and depression is thus extremely relevant considering the treatment options available for the latter, with potential impact on quality of life and survival. We emphasize it is important that clinicians recognize affective symptoms and diagnose depression early in ALS patients.

A meta-analysis of 46 studies investigating overall depression in a total of 7171 ALS patients reported a summary prevalence of depressive symptoms of 34% [83]. Furthermore, in a case–control study including 1752 patients, patients with ALS were at higher risk of receiving a clinical diagnosis of depression and using antidepressants within the first year after diagnosis compared to controls [88]. A consistent relationship between the rates of distress or depression and ALSFRS-R scores was shown in a multicenter cohort assessing 329 ALS patients. In total, 12% of the patients included were diagnosed with depression and recorded lower scores on the total ALSFRS-R, fine motor and gross motor function [6], similar to other study results [84].

Our data support these findings; the results show a negative correlation between the BDI scores and disease severity, and thus indicate that affective symptoms are associated with lower functional performance in our patients. As for the relationship between survival and depression in ALS patients, the severity of depression may influence survival of these patients [7].

Few studies have evaluated non-motor symptoms in ALS patients using standardized scales. In a cohort of 58 patients, Jaafar et al. reported that every individual experienced at least two NMS. Most reported symptoms were fatigue, depression, dysphagia, anxiety and constipation, which closely followed our observations [89].

Our findings show a high burden of non-motor symptoms in ALS patients. The strength of our study consists in the large number of ALS patients included. Moreover, it is the first study conducted on Romanian ALS patients that assesses non-motor symptoms.

This study has several limitations. The data were collected and processed from questionnaires administered through structured interviews, which are more subjective and thus entail validity issues. However, such interviews are accessible and applicable in daily practice, and they also allow clarifications and minimize reporting bias. We agree that future studies should integrate objective clinical assessments (i.e., neuropsychological testing, polysomnography, urodynamic evaluation) to validate and complement patient-reported outcomes.

We also acknowledge the relatively small sample size of both the patient and the control groups, and the challenges inherent in recruiting patients with a rapidly progressive neurodegenerative disease. Moreover, the control group was younger than the study group, this imbalance reflecting the practical challenges of recruiting healthy, age-matched volunteers, particularly in older age groups. The difference in group characteristics is recognized as a limitation, and future studies with larger, age-matched cohorts are warranted.

Another limitation is the ΔPR formula, which was originally validated in prospective cohorts, while in our case they were retrospectively extracted. Although the average time from onset to diagnosis was short, alternative approaches, such as late slope models, may be more appropriate for future studies.

While this study shows a high prevalence of non-motor symptoms in ALS patients, we acknowledge that the observational cross-sectional design limits causal inferences. Moreover, uncorrected *p*-values increase the risk of Type I error so that the associations observed should therefore be interpreted with caution. However, the observed associations between specific NMS and ALFSFRS-R subscores further suggest that these symptoms are not incidental, but most likely reflective of ALS-related pathophysiological processes, and, as emerging evidence suggests, they may influence prognosis and disease trajectory. Future research should aim to clarify the prognostic value of non-motor symptoms in ALS through longitudinal, multicenter studies with larger and more demographically diverse cohorts. Such designs would allow assessment of how specific non-motor features influence functional decline, quality of life, and survival. Integrating objective evaluations (i.e., heart rate variability tests, sleep studies, neuropsychological testing) with patient-reported measures would strengthen the validity of findings and reduce self-report bias. Moreover, the development and validation of ALS-specific instruments would provide a more accurate framework for quantifying the burden of non-motor symptoms.

## 5. Conclusions

The impact of NMS on ALS patient’s lives is profound, extending well beyond the classical motor deficits. Recent advances in ALS research have prompted a paradigm shift, recognizing the disease as a multidimensional disorder encompassing motor, cognitive, behavioral, and systemic manifestations. Expanding research efforts have highlighted the clinical relevance of NMS, and our findings—despite being based on self-reported data from structured interviews and validated questionnaires—offer valuable insight into patients’ lived experiences.

Our study highlights the significant burden of non-motor symptoms in ALS and reveals distinct patterns of association with motor function, affective state and disease progression. Including a healthy control group allowed us to contextualize the BDI and FAB scores observed in patients, enabling the identification of disease-specific alterations in mood and executive function.

To summarize, there are 3 key findings in our study:

1. There is a high prevalence of NMS in our ALS study patients, with the majority of them presenting with more than one non-motor symptom according to the survey results.
2. The presence of NMS is not consistently reported by patients with greater functional impairment, as they do not uniformly correlate with lower ALS functional scores—somatosensory and urinary involvement may occur independently of the extent of motor disability.
3. Affective, cognitive and behavioral changes co-occur with motor symptoms, and they are expected to be more prominent in patients with more severe motor impairment.

In conclusion, broadening the clinical focus beyond motor symptoms is essential for improving diagnostic precision and developing targeted, patient-centered interventions. Systematic identification and management of cognitive, emotional, and behavioral symptoms can support more comprehensive care, ultimately enhancing quality of life. Given the substantial psychological burden on patients and caregivers, a compassionate, multidisciplinary approach should be integrated into standard ALS care.

## Figures and Tables

**Table 1:** Participants demographics and clinical characteristics.

	Patients (N = 44)	HC (N = 35)	*p*
Age (mean ± SD) (years) ^o^	58.39 (±12.52)	53.11 (±9.84)	0.044
Sex ratio (M:F)	28:16	23:12	0.847
BDI (mean ± SD) ^o^	10.75 (±8.59)	5.49 (±3.04)	<0.001
FAB (mean ± SD) ^o^	13.45 (±4.69)	17.46 (±1.01)	<0.001
ALSFRS-R scores ^o^			
Respiratory subscore (±SD)	11.4 (±0.9)	NA	NA
Bulbar subscore (±SD)	10.1 (±2.4)	NA	NA
Fine Motor subscore (±SD)	8.8 (±2.7)	NA	NA
Gross Motor subscore (±SD)	6.3 (±3.4)	NA	NA
Total score	37.6 (±6.3)	NA	NA
ALS subtype ^∞^			
Bulbar	9 (20.5%)	NA	NA
Spinal	35 (79.6%)	NA	NA
ALS phenotype ^∞^			
Classic	25 (56.8%)	NA	NA
LMN	12 (27.3%)	NA	NA
Bulbar	7 (15.9%)	NA	NA
Progression pattern ^∞^			
Horizontal	23 (52.3%)	NA	NA
Vertical	21 (47.7%)	NA	NA
ΔPR (range)	0.5 (0.3–1.3)	NA	NA
Time from onset to diagnosis (months) *	9.5 (5.75–24.25) *	NA	NA

Abbreviation: BDI: Beck Depression Inventory; FAB: Frontal Assessment Battery; HC: healthy controls; LMN: lower motor neuron; ΔPR: disease progression rate; ^∞^ categorical variables reported as frequencies and percentages; ^o^ parametric distribution reported as mean (±SD); * non-parametric distribution reported as median (IQR).Compared to HC, patients had significantly higher BDI scores (10.75 ± 8.59 vs. 5.49 ± 3.04, *p* < 0.001) and lower FAB scores (13.45 ± 4.69 vs. 17.46 ± 1.01, *p* < 0.001), although the mean scores in both groups are within the normal range (BDI cutoff < 13; FAB cutoff > 12).

**Table 2:** Non-motor symptoms questionnaire results.

N = 44	N (%) *
**Cognitive changes**	7 (15.9%)
memory impairment	4.5%
attention/concentration deficit	13.6%
**Psychiatric symptoms**	28 (63.3%)
apathy	18.2%
sadness	56.8%
anxiety	47.7%
perceptual problems	2.3%
hallucinations	0%
**Sleep disorders/fatigue**	35 (79.6%)
tiredness	2.3%
insomnia	72.7%
vivid dreams	65.9%
sleep-talking	11.4%
**Sensory symptoms**	37 (84%)
pain	61.4%
discomfort in legs	77.3%
swollen legs	20.5%
**Cardiovascular symptoms**	30 (68.2%)
dizziness/light-headedness	68.2%
Falls	50%
**Urinary symptoms**	16 (36.4%)
urinary urgency	22.7%
nocturia	31.8%
**Sexual symptoms**	27 (61.4%)
interest in sex	56.8%
sexual dysfunction	56.8%
**Gastrointestinal symptoms**	36 (81.8%)
dribbling	31.8%
difficulty swallowing	34.1%
vomiting/nausea	22.7%
constipation	75%
bowel incontinence	18.2%
incomplete bowel emptying	29.5%
**Miscellaneous**	31 (70.5%)
loss of/change in taste/smell	25%
weight change	31.8%
excessive sweating	13.6%
double vision	0%

* All categorical variables reported as frequencies and percentages.

**Table 3:** ANOVA test for ALSFRS-R scores and ΔPR grouped by non-motor symptoms.

	Mean (±SD)	Mean (±SD)	*p* *
Cognitive changes			
	YES	NO	
ALSFRS-R Respiratory	11.6 (±0.5)	11.4 (±0.9)	0.618
ALSFRS-R Bulbar	8.6 (±2.4)	10.4 (±2.3)	0.062
ALSFRS-R Gross Motor	6.6 (±4.0)	6.2 (±3.4)	0.819
ALSFRS-R Fine Motor	8.9 (±3.3)	8.7 (±2.6)	0.910
ALSFRS-R Total	36.6 (±6.2)	37.8 (±6.4)	0.647
ΔPR	1.0 (±1.9)	0.9 (±0.9)	0.753
Time from onset to diagnosis	29.6 (±20.0)	13.5 (±12.0)	**0.006**
Psychiatric symptoms			
	YES	NO	
ALSFRS-R Respiratory	11.5 (±0.9)	11.2 (±0.9)	0.233
ALSFRS-R Bulbar	9.8 (±2.5)	10.6 (±2.2)	0.322
ALSFRS-R Gross Motor	6.6 (±3.7)	5.8 (±3.0)	0.431
ALSFRS-R Fine Motor	9.1 (±2.6)	8.1 (±2.8)	0.201
ALSFRS-R Total	38.2 (±6.3)	36.5 (±6.5)	0.393
ΔPR	0.9 (±1.1)	1.0 (±0.9)	0.607
Time from onset to diagnosis	18.7 (±15.8)	11.5 (±11.2)	0.117
Sleep disorders			
	YES	NO	
ALSFRS-R Respiratory	11.3 (±0.9)	12 (±0.0)	**0.030**
ALSFRS-R Bulbar	9.7 (±2.5)	11.4 (±0.9)	0.053
ALSFRS-R Gross Motor	6.4 (±3.6)	5.9 (±3.1)	0.695
ALSFRS-R Fine Motor	8.3 (±2.7)	10.7 (±1.4)	**0.014**
ALSFRS-R Total	36.3 (±6.4)	42.4 (±2.9)	**0.008**
ΔPR	1.0 (±1.1)	0.6 (±0.6)	0.317
Time from onset to diagnosis	16.54 (±14.8)	14.22 (±14.4)	0.675
Sensory symptoms			
	YES	NO	
ALSFRS-R Respiratory	11.3 (±1.0)	11.9 (±0.4)	0.164
ALSFRS-R Bulbar	10.0 (±2.5)	10.9 (±1.6)	0.355
ALSFRS-R Gross Motor	6.8 (±3.4)	3.9 (±2.2)	**0.039**
ALSFRS-R Fine Motor	8.5 (±2.8)	10.0 (±1.5)	0.181
ALSFRS-R Total	36.8 (±6.2)	41.6 (±5.5)	0.069
ΔPR	1.0 (±1.1)	0.4 (±0.2)	0.154
Time from onset to diagnosis	17.3 (±14.9)	9.6 (±10.9)	0.202
Cardiovascular symptoms			
	YES	NO	
ALSFRS-R Respiratory	11.2 (±1.0)	11.8 (±0.4)	0.064
ALSFRS-R Bulbar	9.7 (±2.6)	10.9 (±1.7)	0.109
ALSFRS-R Gross Motor	5.8 (±3.6)	7.3 (±2.9)	0.194
ALSFRS-R Fine Motor	8.3 (±2.6)	9.7 (±2.7)	0.103
ALSFRS-R Total	36.2 (±5.8)	40.6 (±6.4)	**0.027**
ΔPR	0.9 (±0.9)	0.9 (±1.3)	0.817
Time from onset to diagnosis	18.9 (±15.2)	9.9 (±11.5)	0.055
Urinary symptoms			
	YES	NO	
ALSFRS-R Respiratory	11.7 (±0.6)	11.3 (±1.0)	0.132
ALSFRS-R Bulbar	11.2 (±1.3)	9.4 (±2.6)	**0.012**
ALSFRS-R Gross Motor	5.6 (±3.2)	6.8 (±3.5)	0.289
ALSFRS-R Fine Motor	8.8 (±2.2)	8.8 (±2.9)	0.908
ALSFRS-R Total	38.8 (±4.9)	36.9 (±7.0)	0.338
ΔPR	0.5 (±0.5)	1.2 (±1.2)	**0.045**
Time from onset to diagnosis	19.1 (±14.7)	14.4 (±14.5)	0.309
Sexual dysfunction			
	YES	NO	
ALSFRS-R Respiratory	11.5 (±0.8)	11.3 (±1.1)	0.518
ALSFRS-R Bulbar	9.8 (±2.6)	10.6 (±1.8)	0.273
ALSFRS-R Gross Motor	6.6 (±3.3)	5.8 (±3.6)	0.475
ALSFRS-R Fine Motor	8.8 (±2.7)	8.7 (±2.8)	0.932
ALSFRS-R Total	37.3 (±6.2)	38.5 (±6.7)	0.702
ΔPR	1.0 (±1.2)	0.8 (±0.7)	0.501
Time from onset to diagnosis	18.7 (±15.7)	11.9 (±11.8)	0.138
Digestive symptoms			
	YES	NO	
ALSFRS-R Respiratory	11.3 (±0.9)	12.0 (±0.0)	**0.044**
ALSFRS-R Bulbar	9.7 (±2.5)	11.8 (±0.7)	**0.026**
ALSFRS-R Gross Motor	6.4 (±3.5)	5.6 (±3.1)	0.547
ALSFRS-R Fine Motor	8.4 (±2.8)	10.1 (±1.8)	0.109
ALSFRS-R Total	36.5 (±6.4)	42.5 (±3.2)	**0.013**
ΔPR	0.9 (±1.1)	0.6 (±0.8)	0.341
Time from onset to diagnosis	17.1 (±14.4)	11.6 (15.5)	0.347

* Statistically significant variation set at *p* < 0.05.

## Data Availability

The data presented in this study are available on request from the corresponding author but will not be publicly available due to restrictions from our funding contract.
